# *Mu killer*-Mediated and Spontaneous Silencing of *Zea mays* Mutator Family Transposable Elements Define Distinctive Paths of Epigenetic Inactivation

**DOI:** 10.3389/fpls.2012.00212

**Published:** 2012-09-13

**Authors:** David S. Skibbe, J. F. Fernandes, Virginia Walbot

**Affiliations:** ^1^Department of Biology, Stanford UniversityStanford, CA, USA

**Keywords:** transposon, mutator, *Mu killer*, anther, maize, splicing

## Abstract

*Mu killer* contains a partial inverted duplication of the *mudrA* transposase gene and two copies of the terminal inverted repeat A (TIRA) region of the master *MuDR* element of maize. *Mu killer* can effectively silence single copy *MuDR/Mu* lines, and it is proposed that a ∼4 kb hairpin RNA is generated by read through transcription from a flanking gene and that this transcript serves as a substrate for siRNA production. *Mu killer* was sequenced, except for a recalcitrant portion in the center of the locus, and shown to be co-linear with *mudrA* as originally proposed. The ability of the dominant *Mu killer* locus to silence a standard high copy number *MuDR/Mu* transposon line was evaluated. After two generations of exposure, about three quarters of individuals were silenced indicating reasonable effectiveness as measured by the absence of *mudrA* transposase transcripts. *Mu killer* individuals that effectively silenced *MuDR* expressed two short antisense transcripts. In contrast, *Mu killer* individuals that failed to silence *MuDR* expressed multiple sense transcripts, derived from read through transcription initiating in a flanking gene, but no antisense transcripts were detected.

## Introduction

*MuDR/Mu* comprise the mutator family of maize Class II transposable elements (reviewed in Lisch, 2002; Walbot and Rudenko, 2002). These high copy number elements are very effective mutagens, because they target RNA Polymerase II transcription units (Fernandes et al., 2004); *MuDR/Mu* in active mutator lines increase the forward mutation frequency 50–100-fold per locus (Walbot and Rudenko, 2002). *MuDR* contains two convergently transcribed genes: *mudrA* encodes transposase and *mudrB* encodes a protein of unknown function. Transcripts of both genes are subject to alternative splicing, which results in multiple protein isoforms (Walbot and Rudenko, 2002). The diverse elements share ∼215 bp terminal inverted repeats (TIRs) within which there is a highly conserved MURA transposase binding site in all mobile *Mu* (Benito and Walbot, 1997) and the gene promoters (Raizada et al., 2001a,b). A key feature of the family is the high copy number of ∼50–100 mobile *Mu* elements in active mutator plants. Utilizing replicative transposition in which parental transposon locations are preserved, *MuDR/Mu* elements double in copy number late in the lifecycle by inserting throughout the genome. Consequently, progeny of an outcross to a non-mutator line have about the same number of elements as the mutator parent (Alleman and Freeling, 1986; Walbot and Warren, 1988).

When *MuDR/Mu* copy number increases substantially, as after self-pollination, the likelihood of transposon silencing increases (Robertson, 1986; Walbot, 1986). Historically *Mu* element silencing was followed using visible phenotypes, either loss of somatic excision from mutable alleles, for example the switch from spotted to colorless kernels (Robertson, 1986; Walbot, 1986), or by activation of gene expression at a locus suppressed by a nearby *Mu* insertion, for example converting pale green to darker leaf pigmentation (Martienssen et al., 1990). Molecular assays for silencing include DNA methylation within *Mu* elements (Chandler and Walbot, 1986) and decreased *mudrA* transposase transcripts (Rudenko et al., 2003). Although some individuals switch abruptly from an active to inactive status, in many cases loss of *Mu* element insertion and excision occur over a span of time. Silencing is progressive, both within the sequential organs of a developing plant (Martienssen et al., 1990) and over several generations (Takumi and Walbot, 2007). The trigger for silencing other than the correlation with high *MuDR/Mu* copy number has been difficult to define, although there are clearly separable steps for imposition and maintenance of silencing (Woodhouse et al., 2006).

**Figure 1:**
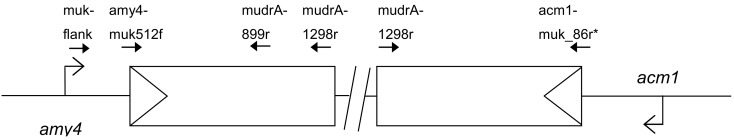
**Schematic of the *Mu killer* locus and the two flanking maize genes *amy4* and *acm1***. The triangles indicate the TIRA sequences. Primers used in the analysis of *Mu killer* genomic DNA and RT-PCR products are indicated.

The stochastic appearance and incomplete penetrance of silencing have been barriers to establishing the specific components of the silencing machinery. All maize lines contain defective *hMuDR* elements (Rudenko and Walbot, 2001) that generate both sense and antisense transcripts, however, these elements are insufficient to direct silencing efficiently in crosses with active mutator individuals. Similarly introduction of antisense transgenes is not an efficient silencing method (Kim and Walbot, 2003). In an unusual single copy *Mu1* plus single copy *MuDR* line, called minimal mutator (Lisch et al., 1995), spontaneous silencing is exceedingly rare (Lisch and Freeling, 1994). This permitted detection of a newly arisen dominant factor, *Mu killer* (GenBank accession: DQ011286), that could swiftly silence the minimal mutator system (Slotkin et al., 2003). The *Mu killer* locus has an internally deleted and partially duplicated *MuDR* element (Figure 1); the two TIRs are both derived from the *mudrA* adjacent element end and there are two partial copies of *mudrA* that terminate in an inverted duplication. The precise DNA sequence of *Mu killer* has not been reported. It is hypothesized that transcripts containing the inverted *mudrA* duplicated region form fold back RNA that is processed into small interfering RNAs that direct silencing of the single *MuDR* in the line (Slotkin et al., 2005).

We have reported a transcriptomic and proteomic comparison of anthers from sister lines that remained active or were recently fully silenced. In standard, high copy number active mutator lines the transcriptome is altered ∼25% in comparison to the silenced line, including activation of stress pathways (Skibbe et al., 2009), indicating that mutator activity has a major impact on the host without disrupting normal anther development. In the current study we sought to determine if *Mu killer* could silence a standard high element copy number mutator line, because these lines are non-responsive to ectopically expressed antisense RNA (Kim and Walbot, 2003). Second, if *Mu killer* can invoke silencing in such lines is the impact on the transcriptome similar to what occurs during spontaneous silencing? Third, we wished to better define the structure of *Mu killer* as an aid to understanding the structure of predicted RNA transcripts that might be mechanistically involved in *MuDR/Mu* silencing.

## Materials and Methods

### Genetic materials

**Figure 2:**
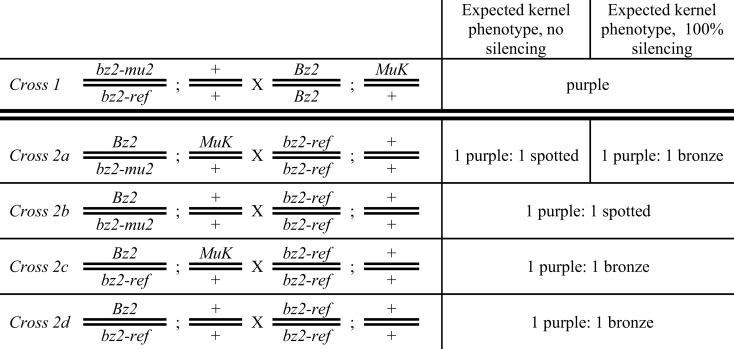
**Crossing scheme to test for *Mu killer*-induced silencing of a high copy mutator line**. Half of the progeny ears contain the bz2-mu2 reporter allele and are expected to segregate 1:1 purple:spotted (Crosses 2a and 2b); the other half (Crosses 2c and 2d) lack the reporter allele and are expected to segregate 1:1 purple:bronze. Mu killer is segregating 1:1 in Crosses 2a and 2c.

A *Mu killer*, *a1-mu1//a1* single copy *MuDR* minimal mutator stock was obtained from Damon Lisch. This line was maintained by crossing to an *a1* minimal mutator line or to an *a1* tester grown at Stanford CA or on Moloka’i HI. High copy *MuDR/Mu* lines with *bz2-mutable* alleles were described previously (Skibbe et al., 2009). The crossing program for evaluating the capacity of the *Mu killer* line to silence the high copy number lines is presented in Figure 2.

### Sequencing *Mu killer*

The *Mu killer* locus was PCR amplified in two halves (Figure 1). A 1.8 kb amplicon was obtained for the “left” half using the muk-flank (5′ CCTTTGGTCAGTTCGGTTATCTCTG 3′) and *mudrA*-1298r (5′GCACCCATTTGTGGTTTCTT 3′) primers. A 1.3 kb amplicon was obtained for the “right” half using the *acm1-Mu killer* chimeric primer acm1-muk_86r (5′ CCCCTACTTGTTGTTGAGATAATTG 3′) and mudrA-1298r primer. Genomic DNA targets were amplified in a 25 μl reaction using 20 ng genomic DNA as template, 0.5 μM of each primer, 2 mM MgSO_4_, 0.2 mM dNTPs, 1× *HiFi* Buffer, and 1 unit *HiFi Platinum Taq* (Invitrogen Life Science, Carlsbad CA, USA) per reaction under the following conditions: 94°C for 2 min followed by 35 cycles of 94°C for 30 s, 60°C for 45 s, and 72° for 1.5 min; there was a final extension at 72°C for 10 min. The left and right *Mu killer* amplicons were purified using the Qiagen (Valencia, CA, USA) PCR Purification Kit, TOPO subcloned, and sequenced using M13 forward and M13 reverse primers. The *Mu killer* sequence segments were assembled and validated by replicate sequencing and are available under GenBank numbers JX067398 (left half) and JX067399 (right half).

### Genotyping

Genomic DNA was extracted from adult leaf tissue using the CTAB method of Rogers and Bendich (1985). Genotyping was performed using the PCR parameters described above. Target, amplicon size, and corresponding primer pairs are: *Mu killer*, 0.7 kb amplicon with the muk-flank (5′ CCTTTGGTCAGTTCGGTTATCTCTG 3′) and mudr-219r (5′ AGGAGAGACGGTGACAAGAGGAGTA 3′) primers; *bz2-mu2*: 0.2 kb amplicon with the bz2-637f (5′ CAGAGAGGTGCCAACAGAAGT 3′) and Mu-TIR (5′ AGAGAAGCCAACGCCAWCGCCTCYATTTCGTC 3′) primers.

### RNA analysis

Total RNA was extracted from either whole spikelets containing 1 mm (pre-meiotic) to ∼2 mm (late prophase I meiotic stage) anthers or dissected 1–2 mm anthers using Trizol reagent (Invitrogen) according to the manufacturer’s instructions. RNA quantity and quality were assessed with the Quant-iT RiboGreen RNA Assay Kit (Invitrogen) and agarose gel electrophoresis, respectively. Approximately 1 μg of total RNA was treated with DNase I and reverse transcribed into cDNA with an oligo(dT) primer using the SuperScript III Reverse Transcription system (Invitrogen) as per manufacturer’s instructions. Approximately 20 ng cDNA was used as template for RT-PCR. Target, expected amplicon size, and corresponding primer pairs are: *Mu killer*, 0.5 and 0.7 kb amplicons with the muk-flank (5′ CCTTTGGTCAGTTCGGTTATCTCTG 3′) and mudr-219r (5′ AGGAGAGACGGTGACAAGAGGAGTA 3′) primers; 0.5 kb (spliced) and 0.6 kb (alternatively spliced) *mudrA* with the mudrA-2345f (5′ AGGAATGGCAACACACTGGGAAAC′ 3′) and mudrA-2938r (5′ TTCAGTGACTTCCTCTGCTACGTC 3′) primers; and *tubulin6* (a DNase I control transcript), 0.5 kb (cDNA) with the tub6-475f (5′ AGTATGCCACTCCCTTGGTG 3′) and tub6-979r (5′ GGCACACATCATGTTCTTGG 3′) primers. Other primers used to amplify *Mu killer* were: amy-muk512f (5′AGTCCAGTCCGAGATAATTGC 3′) and mudrA-899r (5′ TTGTCCGTATCCAAACTTCCCT 3′). PCR amplification parameters were identical to those described above. PCR products were resolved on a 2% agarose/TBE gel stained with ethidium bromide.

### Transcriptome profiling

Individuals from ears segregating for mutator active and siblings silenced by *Mu killer* were used as generated from Cross 2 (Figure 2). Spotted (mutator active) or bronze (*Mu killer*) kernels were selected and grown in Stanford, California in summer 2008, and the presence or absence of *Mu killer* was confirmed via PCR on genomic DNA. Anthers were staged from the upper florets and then dissected onto dry ice and stored at −80°C. Total RNA was extracted from four biological replicates of mutator active and *Mu killer* anthers (15–20 anther sample size) and quantified and qualitatively assessed as described above. DNase I-treated total RNA (1 μg) was amplified and labeled with either Cy3 or Cy5 using a Low RNA Input Fluorescent Linear Amplification Kit (Agilent Technologies, Santa Clara, CA, USA). Eight hundred twenty-five nanograms of each labeled cRNA was hybridized for 17 h at 60°C on a 4 K × 44 K Agilent *in situ* synthesized oligonucleotide platform along with spike-in controls for calculating target RNA concentrations. Data acquisition, image processing, spot flagging and removal, and normalization were performed as described in Skibbe et al., 2009. Probes were considered “on” if fluorescence values were greater than a cutoff of 2.6 SD above the average background for all four replicates. Probes with only three replicates above the cutoff were also considered “on” if the average intensity was above the median for the set of probes with only three replicates above the cutoff. All microarray data associated with these experiments are available at GEO^1^ under the series accession number GSE38314.

## Results

### *Mu killer* transcriptional silencing of *MuDR* in high copy lines is relatively efficient but incomplete

*Mu killer* acts as a dominant locus that effectively silences the minimal mutator system (Slotkin et al., 2003); based on the presence of a large inverted duplication of ∼1300 bp of the *mudrA* gene (Figure 1), it is hypothesized that *Mu killer* encodes a large fold back, double-stranded RNA transcript directing *MuDR* silencing by targeting transposase-encoding *mudrA* transcripts. *Mu killer* effectiveness in standard, high copy number mutator lines has not been reported. To address this question, a line with a single copy of *Mu killer* in a *Bz2* line was crossed (Cross 1) to a high copy mutator *bz2-mu2*//*bz2-ref* spotted kernel line that has been demonstrated to have both a high forward mutation frequency and a large impact on the transcriptome of developing maize anthers (Skibbe et al., 2009). When the mutator system is active, late somatic excision from the *bz2-mu2* reporter allele results in fine purple spots on a beige (bronze) background; when mutator activity is silenced, kernels are uniformly bronze. After Cross 1 all progeny have purple seed, requiring genotyping to identify the *Mu killer* carriers. As expected, all these individuals were also carriers of the *MuDR/Mu* transposon system. Because *Mu killer-*mediated silencing occurs most effectively when *Mu killer* is inherited through the female parent, an additional cross was performed by a *bz2* tester strain. If *Mu killer* is highly effective, then the expectation was that half of the progeny ears would segregate 1:1 purple:bronze and half of the progeny ears would be fully bronze. Among the fully bronze cohort, there are four classes. Two of the classes lack the *bz2-mu2* reporter gene and will be bronze only, independent of *Mu killer*. Classes 2a and 2b (Figure 2) inherit the *bz2-mu2* reporter allele; both of these classes were exposed to *Mu killer* in the previous generation and one class retains *Mu killer* (Class 2a, segregating 1:1 in the progeny).

In Crosses 2a and 2b, ears contained the expected 50% purple kernels, but the remaining kernels were a mixture of spotted (active mutator) and bronze (loss of mutator activity). The mutator system can silence spontaneously, and there is a very wide variation in this frequency and in the percentage of spotted kernels during progressive spontaneous silencing (Takumi and Walbot, 2007). Therefore, interpreting loss of the somatic kernel phenotype is problematic. Instead, we conducted a molecular test independent of the spotted kernel phenotype to assess the ability of *Mu killer* to eliminate *mudrA* transcripts. Plants were grown from bronze kernels of Cross 2a (*Mu killer* carrier parent), using genotyping to pick plants that inherited *Mu killer*, to compare to plants derived from spotted kernels of the parental *bz2-mu2/bz2* active mutator line. Anthers were chosen for analysis because they are the final organs differentiated and thus represent the location most likely to experience silencing.

**Figure 3:**
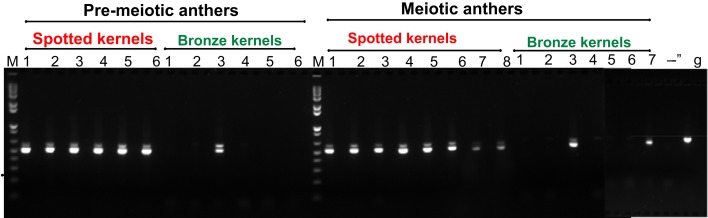
**RT-PCR assessment of *mudrA* transcripts from *Mu killer* and *Mu* active individuals**.

The selected primers span the third intron of *mudrA*; this region of *MuDR* is absent from the *Mu killer* locus. The primers amplify a single genomic DNA band (Figure 3, lane g); however, near-complete splicing of the third intron, as expected (Hershberger et al., 1995), generated a smaller cDNA product with a faint larger band in active mutator samples. RT-PCR confirmed that all spotted kernels from the standard mutator line resulted in plants that amplified this 3′ region of *mudrA* transcripts indicative of maintenance of active mutator status; note that individuals 7 and 8 in the meiotic anther samples contain lowered *mudrA* transcript, an indication of higher likelihood of spontaneous loss of mutator activity (Rudenko et al., 2003). In contrast, only 3/13 of the *Mu killer* carrier bronze kernel plants retained *mudrA* transcripts (pre-meiotic anther sample 3 and meiotic anther samples 3 and 7). These initial results are consistent with *Mu killer* acting as a reasonably efficient silencing agent of high copy mutator lines when present for two generations of plant growth.

### Attempts to fully sequence the *Mu killer* locus

The structure of the *Mu killer* transcript has been proposed to be a hairpin ∼4 kb in length, with an inversion point ∼1.3 kb into the *mudrA* region of the *MuDR* transposon. The structure of the *Mu killer* locus is based primarily on Southern blot evidence. To examine the proposed structure in more detail, the *Mu killer* locus was amplified using primers that are specific to *mudrA*, the TIR adjacent to *mudrA*, and the flanking *amy4* and the *acm1* genes to validate the gene order. The left and right halves of *Mu killer* were amplified in contiguous molecules of 1.8 and 1.3 kb, respectively, and fully sequenced and deposited into GenBank. The *Mu killer* locus begins with terminal inverted repeat A (TIRA) of *MuDR* and extends 1.298 kb into the *mudrA* gene on both the left and right halves. PCR amplification attempts using primers internal to 1.298 kb were unsuccessful indicating that the central region of *MuDR* is not present. Attempts to span the two halves using long-range PCR methods also failed; this result raises the possibility that an insertion/deletion polymorphism, a large rearrangement of unknown origin, or an exceptionally stable DNA hairpin structure between the left and right halves of the *Mu killer* locus prevents PCR amplification.

### Unexpected transcripts encoded by *Mu killer*

In their analysis of the *Mu killer* locus, Slotkin et al. (2005) proposed novel transcription regulation in which the promoter of one of the flanking genes drives expression of *Mu killer* transcripts proposed to cause mutator silencing. This model invokes the unexpected feature that both TIRA promoters are inactive and that the TIRs fail to “insulate” the element from read through transcription.

**Figure 4:**
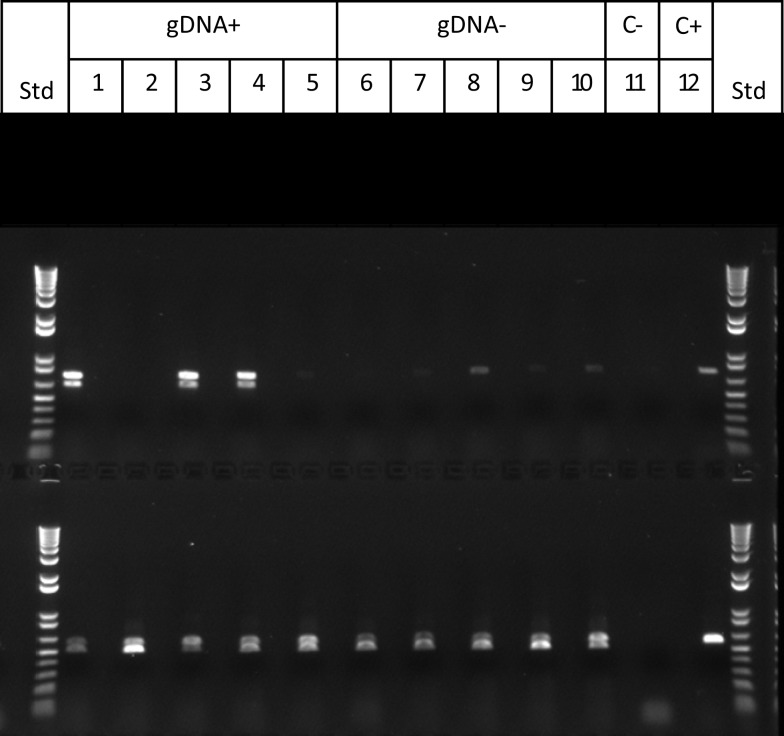
***Mu killer* transcript accumulation in whole spikelets**. The upper panel primer pair amplifies *Mu killer;* the lower panel primer pair amplifies the 3′ end of *MuDR* (not present in *Mu killer*). Lanes 1–5 are five *Mu killer* gDNA “+” individuals; Lanes 6–10 are five *Mu killer* gDNA “−” individuals; Lane 11 is the negative PCR control; Lane 12 is the positive PCR control.

To test these aspects of the model, progeny from Cross 1 were classified as active (gDNA “+”) or inactive (gDNA “−”) in silencing based on PCR confirmation of *Mu killer* using genomic DNA. In an effort to identify *Mu killer* transcripts associated with silencing activity, five gDNA “−” and five gDNA “+” individuals were tested via RT-PCR for accumulation of *MuDR* and *Mukiller*-derived transcripts in whole spikelets. As expected, all of the gDNA “−” individuals accumulated *MuDR*-derived transcript but failed to amplify detectable levels of *Mukiller* transcript (Figure 4, lanes 6–10). Surprisingly, only three of the five gDNA “+” individuals (Figure 4, lanes 1, 3, and 4, top row) amplified *Mu killer* transcript types. Furthermore, although only a 0.7 kb transcript was previously reported, an additional type was detected here. The remaining two gDNA “+” individuals (Figure 4, lanes 2 and 5, top row), failed to amplify detectable levels of *Mukiller* transcript. The *MuDR* and *tubulin 6* control primers amplified as expected for each individual.

**Figure 5:**
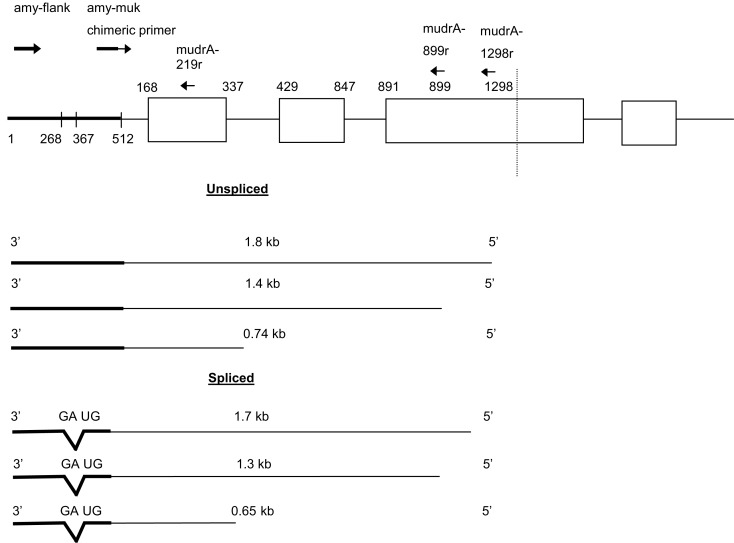
**Antisense transcripts initiating in *Mu killer* and continuing into *amy4* occur in plants with *Mu killer*-induced mutator silencing**. Individuals were scored as having experienced *Mu killer*-induced silencing based on having genomic DNA positive for *Mukiller* but cDNA that lacked *mudrA* 3′ transcripts indicative of mutator activity (Figure 1).

**Figure A1:**
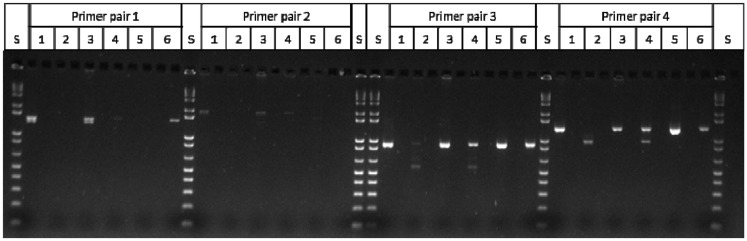
***Mu killer* individuals undergoing *Mu killer* silencing accumulate aberrant *Mu killer* transcript types**. *S* = 1 kb plus DNA ladder; Lanes 1–4 = *Mu killer* gDNA “+” individuals; Lanes 1 and 3 accumulate expected transcript types; Lanes 2 and 4 accumulate aberrant transcript types; Lane 5 = mudrA control DNA; Lane 6 = *Mu killer* control DNA; Primer Pair 1: amy4-flank + mudrA-899r (expected size = 1400 bp); Primer Pair 2: amy4-flank + mudrA-1298r (expected size = 1819 bp); Primer Pair 3: amy4-muk_512f + mudrA-899r (expected size = 910 bp); Primer Pair 4: amy4-muk_512f + mudrA-1298r (expected size = 1308 bp). The amy4-muk_512f primer is a chimeric primer that is expected to amplify both *Mu killer* and MuDR loci.

The two individuals that contained *Mu killer* but lacked *Mu killer* cDNA, along with two individuals that did accumulate *mudrA* cDNA were selected for further analysis along with positive and negative controls. The transcript accumulation in these four individuals was interrogated using primers positioned at four locations: (1) ∼500 bp upstream of *Mukiller* (amy4-flank) (2) at the junction of the *amy4*/*Mu killer* (amy4-muk_512f), (3) ∼900 bp into the *Mu killer* gene (mudrA-899r), and (4) ∼1300 bp into the *Mu killer* gene (mudrA-1298r). When the amy4-flank primer was paired with the two internal *Mu killer* primers, amplicons of the expected size (plus a splice variant ∼100 bp smaller) accumulated in the two *Mu killer* transcript “+” individuals but no transcripts were detected in either of the *Mu killer* transcript “−” individuals. When the *amy4*/*Mu killer* chimeric primer was paired with the two internal *Mukiller* primers, amplicons of the expected size (without any splice variants) accumulated in the two *Mu killer* transcript “+” individuals. In the *Mu killer* transcript “−” individuals, amplicons of an unexpected size were detected (Figure A1 in Appendix for the RT-PCR results). All amplicons were purified, sequenced, and the results are diagrammed in Figure 5.

Several unexpected molecular observations were made while assessing accumulation of *Mu killer* and *mudrA* transcripts in whole spikelets and anthers of these effective silencing cases. First, two families of antisense transcripts initiating within *Mu killer* and proceeding toward the *amy4* locus accumulated in whole spikelets (Figure 5). A 0.74 kb amplicon was reported previously (Slotkin et al., 2005); however, none of the larger amplicons or the smaller 0.65 kb size class has been described. Approximately 80% of total transcript is in the 0.65 kb class, therefore, the 0.65 and 0.74 kb transcripts were cloned and sequenced to elucidate their structures. The 0.74 kb transcript type is antisense to *mudrA*. It is co-linear with an internal region contained in both *Mu killer* and *MuDR* (Hershberger et al., 1995) and with TIRA; the transcript terminates within *amy4* (at position 1, Figure 5). Therefore, we conclude that the 0.74 kb transcripts initiate at a previously unreported antisense promoter within the *mudrA* sequence (within the sense intron 1) shared by *Mu killer* and *MuDR*. Additional analysis using RT-PCR amplification employing oligonucleotides further into *Mu killer* (mudrA-899r and mudrA-1298r) demonstrated that longer transcripts (Figure 5) can originate at least 1.298 kb into the *Mu killer* locus, starting within or beyond the region of *Mu killer* that could not be sequenced; it is possible that some transcripts initiate within the right side TIRA of *Mu killer* producing sense *mudrA*-containing RNA (5′ half of the transcripts) plus antisense *mudrA* (3′ half) in the region shared with the 0.74 kb transcript type.

Interestingly, the major 0.65 kb transcript results from splicing of a short intron with canonical 5′ donor and 3′ acceptor sites in the *amy4* flanking region. Similarly, the minor 1.8 and 1.4 kb transcripts diagrammed in Figure 5 are inferred to be unspliced forms, and the minor 1.7 and 1.3 kb forms involve removal of the same intron documented in the 0.65 kb transcripts. Tissue-specific variation in the ratio of the 0.74 kb and alternatively spliced 0.65 kb transcripts was observed in whole spikelets (containing the vegetative glumes, lemma, and palea plus anthers), in anthers, and in adult leaves but the 0.65 kb form is always predominant.

**Figure 6:**
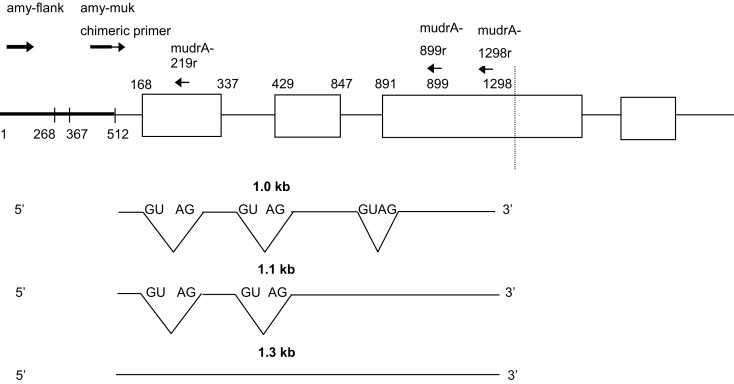
**Sense transcripts contained within *Mu killer* occur in lines failing to silence *MuDR***. The two plants analyzed were genotyped as *Mu killer* carriers, however, the cDNA is negative for *Mu killer* antisense transcripts and positive for *mudrA* sense transcripts covering the 3′ end of the gene characteristic of *mudrA* (Figure 1).

Next, transcripts were analyzed from two individuals that failed to silence *MuDR*: they contained *Mu killer* but generated *mudrA* cDNA. In these individuals, no antisense transcript types were identified that extended into the *amy4* flanking primer region, that is, both the 0.74 and 0.65 kb transcript types were missing. We therefore propose that production of the antisense 0.74 and 0.65 kb transcripts is required for *Mu killer*-mediated silencing of *mudrA*. In individuals that failed to silence mutator, RT-PCR amplification products were only identified for the combination of the chimeric *amy4*/*Mu killer* primer paired with the two internal *mudrA* oligonucleotides (Figure 6). These amplicons are in the sense orientation relative to *mudrA* but were shorter in length than canonical *mudrA* transcripts. Sequence analysis demonstrated that several alternatively spliced transcript types are present; analysis of the acceptor and donor sites confirmed that these transcripts are in the sense orientation with respect to the normal *mudrA* transcript. The introns identified are not spliced from normal *mudrA* transcripts, although alternative splicing does occur in master element transcripts (Hershberger et al., 1995).

Collectively, transcript analyses demonstrated that *Mu killer* individuals that silence *MuDR* accumulate *mudrA* antisense transcripts that extend into the *amy4* flanking gene region; the promoter for these transcripts is not yet defined but it resides inside *Mu killer*. This result disproves the proposed Slotkin et al. (2005) model that antisense transcripts would originate from read through transcription from flanking maize genes. The results also demonstrate that TIRA lacks a transcription terminator in the context of the *Mu killer* structure at this locus. In contrast, *Mu killer* individuals that fail to silence *MuDR* accumulate sense transcript types that are predominately alternatively spliced compared to authentic *mudrA* transcripts. These sense transcripts appear to arise from transcriptional read through from the *amy4* locus, as opposed to the Slotkin et al. (2005) model where antisense transcripts derive from flanking gene promoters. In our analysis such transcripts were determined to be in the sense orientation and not involved in mutator silencing. The existence of read through transcripts indicates that the TIRA in the context of *Mu killer* fails to insulate the *Mu* element from flanking gene transcriptional activity.

### *Mu killer* and spontaneous silencing result in distinctive gene expression changes in anthers

**Figure 7:**
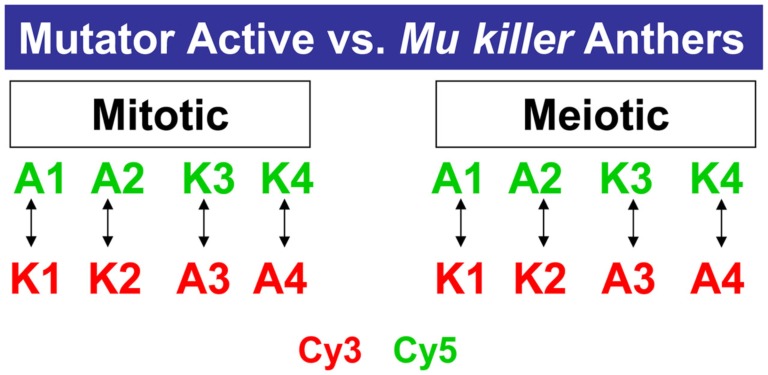
**Microarray profiling experimental design**. Four biological replicates of *Mu* active (A) or *Mu killer* (K) maize anthers were collected for two stages. For each stage, two samples were labeled with the Cy3 fluor (Green) or Cy5 fluor (Red) and hybridized as shown on the 4 K × 44 K Agilent maize oligonucleotide array platform.

A third question we addressed is how similar the consequences are of *Mu killer-*mediated compared to spontaneous epigenetic silencing in anther development. In a previous study, 1 and 2 mm anthers were shown to express more than 30,000 transcripts (Skibbe et al., 2009). In the current study the same criteria to be considered expressed or “on” were employed after analyzing four biological replicates of the mitotic (1 mm) and meiotic (2 mm) anther stages in *Mu* active and *Mu killer* silenced lines (Figure 7).

**Figure 8:**
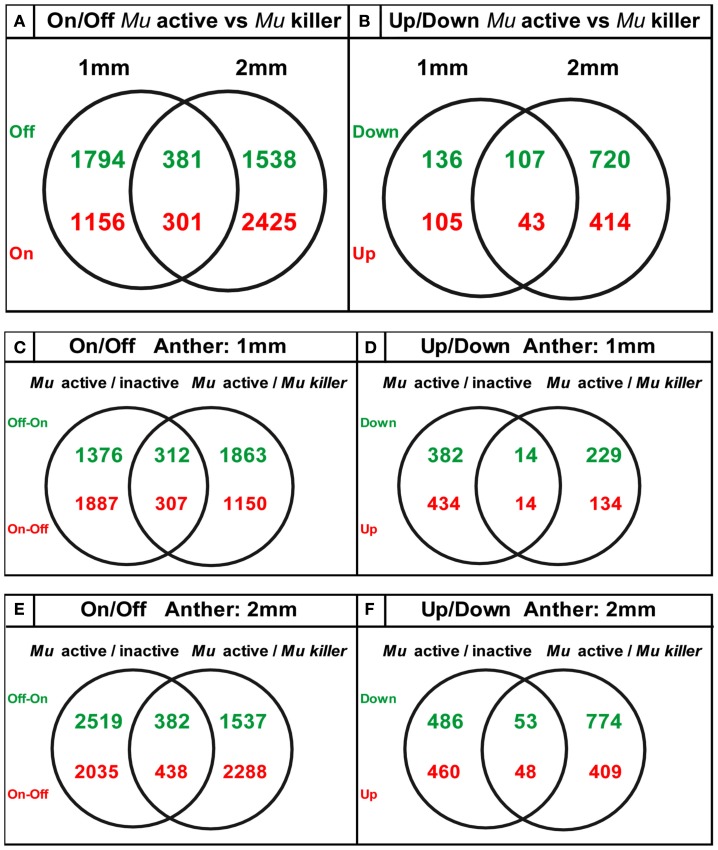
**Venn diagrams showing unique sets of differentially expressed transcripts result from epigenetic and genetic inactivation of the mutator transposon family**.

We found that the pre-meiotic 1 mm anther transcriptome contains 34,338 transcripts in an active mutator background and slightly more (35,056) transcript types in *Mu killer*. The 2 mm meiotic anther samples expressed a similar number of transcripts with 34,508 (*Mu* active) and 33,700 (*Mu killer*). There were many examples of stage-specific transcripts in both biological sample types. Two types of differences in transcript expression comparing *Mu* active to *Mu killer* silenced lines were analyzed: (1) “Off/On” differences represent expression that was detected in one sample but not in the other and (2) “Up/Down” differences are the differential expression class and include probes where expression was at least 1.5-fold higher in one sample than the other (*p-*value < 0.05). Venn diagrams summarizing On/Off differences for *Mu* active vs. *Mu killer* backgrounds for both anther stages are shown in Figure 8. Both stages have distinct sets of more than 1500 transcripts not detected in the *Mu* active background but expressed in *Mu killer* (green numbers).

The ectopic activation of gene expression indicates that *Mu killer* has a major impact on the anther transcriptome compared to an active mutator line. *Mu killer* impact is dynamic, because only 381 transcript types were OFF in *Mu* active anthers at both developmental stages. In the reverse comparison (present in *Mu* active but absent in *Mu killer*) there are 1156 transcripts at 1 mm and 2425 at 2 mm with 301 transcripts absent in *Mu killer* for both anther lengths. Collectively, the differences between the two lines are more than 10% of the anther transcriptome. It is already established that high copy mutator lines express a diverse suite of stress response genes, compared to silenced sister lines (or to similar stage fertile anthers in a standard inbred line; Skibbe et al., 2009). Although not the largest GO category, 29 of the 1457 transcript types present in *Mu* active but missing from *Mu killer* at 1 mm and 47 of the 2726 transcript types at 2 mm are stress-associated. Surprisingly, there were far fewer differentially expressed transcript types than transcripts present in the On/Off category (Figures 8A,B). At 1 mm less than 1% of the transcriptome was impacted in the differential gene expression analysis, with only 2% altered at the 2 mm stage. It appears, therefore, that most transcriptome changes in *Mu* active compared to *Mu killer* lines involve complete suppression or *de novo* activation of suites of genes rather than modulation of transcript levels.

The transcriptome data was next analyzed with regard to how similar epigenetic silencing is to the silencing directed by *Mu killer*. We compared the differentially expressed transcripts determined from the previous study of *Mu* active vs. *Mu* inactive lines (Skibbe et al., 2009) with the differentially expressed transcripts found here in the *Mu* active vs. *Mu killer* lines. For both On/Off and Up/Down differences (Figures 8C + E,D + F) distinctive sets of transcripts are affected at each stage with a much smaller number of transcripts affected in common. These data suggest that *Mu killer* inactivation reprograms gene expression using a different route and perhaps involving different mechanisms that result in such distinctive consequences compared to spontaneous epigenetic silencing.

**Figure 9:**
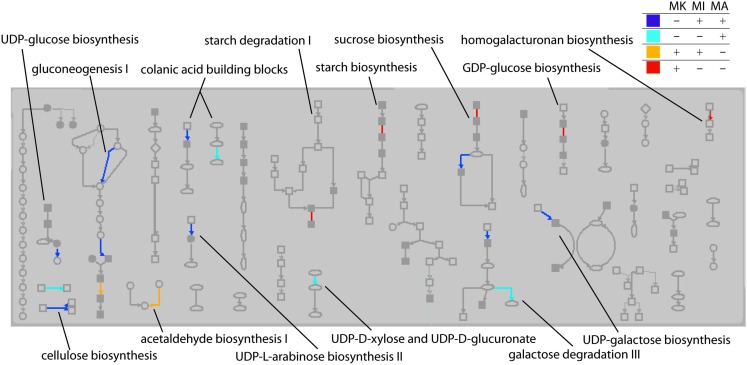
**Carbohydrate biosynthesis pathway class showing reactions affected by *Mu killer* (MK) On/Off transcripts compared to *Mu* inactive (MI) and *Mu* Active (MA)**. Reactions in a pathway that were affected only by *Mu killer* are blue (“off” in *Mu killer*) or red (“on” in *Mu killer*). Reactions affected by both *Mu killer* and *Mu* inactive but not *Mu* active are either teal (“off” in *Mu killer*) or orange (“on” in *Mu killer*).

The transcripts that were “on” in *Mu killer* but “off” in *Mu* active lines at the 2 mm stage, as well as transcripts in the reverse situation, were mapped to version 2 of the MaizeCyc metabolic pathways database^2^ using the Omics Viewer feature of the Pathway Tools application^3^. The Omics Viewer displays metabolic pathways grouped into pathway classes (e.g., carbohydrate cycles or secondary metabolites biosynthesis) to acquire a general picture of the experiment effects. The On/Off status of the *Mu killer* line as compared to the *Mu* inactive line was used to color-code the affected reactions in each pathway; an opposite status is indicated by blue (“off” *Mu killer*, “on” *Mu* inactive) or red (“on” *Mu killer*, “off” *Mu* inactive) and the same status by teal (both “off”) or orange (both “on”). Affected pathways within a sample of five pathway classes were then categorized by their color scheme. As seen in Table 1, the majority of affected pathways were those with transcripts “off” in *Mu killer* but “on” in *Mu* inactive (blue). The combined “different” effects (27, blue and red) is more than the combined “same” effects (20, teal and orange), again indicating that *Mu killer* has distinctive aspects to its silencing mechanism. The many pathways affected in the “Carbohydrate biosynthesis” pathway class are displayed in Figure 9; five pathways have “off” reactions only with *Mu killer* (blue) and four pathways have “on” reactions only in *Mu killer* (red).

**Table 1:** **Five metabolic pathway classes showing affects by *Mu killer* in common or different from *Mu* inactive lines**.

Pathway class	One-color pathways				Two-color pathways			
	Blue	Teal	Red	Orange	BT	RO	BR	BO
Secondary metabolites biosynthesis	10	1	0	1	0	0	0	0
Cofactors, prosthetic groups, electron carriers	2	3	0	2	1	1	1	0
Amino acid biosynthesis	3	1	1	2	0	0	1	1
Carbohydrate biosynthesis	5	3	4	1	1	0	1	1
Hormone biosynthesis	2	4	0	2	0	0	0	0
Totals	22	12	5	8	2	1	3	2

Interestingly, three of the pathway classes in Table 1 had pathways with multiple reactions affected in different ways; these are shown by non-zero counts in the last four columns. A web page was generated from the Pathway Tools with the full Omics Viewer display and is available at http:/www.stanford.edu/~walbot/maizecyc/mukiller_onoff.html. These insights can guide future work in metabolomics and proteomics to discover whether the transcriptomic differences reported here result in distinctive cellular properties.

## Discussion

This study was formulated to examine three aspects of *Mu killer*: effectiveness in silencing standard high *Mu* copy number lines, molecular organization and expression of the locus, and impact on the anther transcriptome. After two generations of *Mu killer* exposure, 77% (10/13) high copy number *MuDR/Mu* individuals tested were silenced based on loss of transcripts characteristic of *mudrA*. Thus *Mu killer*-mediated silencing is considerably more frequent than the spontaneous ∼10–20% loss of mutator activity per generation generally observed (Robertson, 1986; Walbot, 1986) and is a useful practical tool to accelerate mutator silencing. Second, sequencing *Mu killer* confirmed the overall structure originally proposed without resolving the nature of the element center, which failed to amplify in any PCR strategy attempted. On the other hand, detailed analysis of transcript types documented numerous additional transcript types, clarified the strandedness of these and a previously documented transcript, discovered an association between two short antisense transcripts and mutator silencing, and found accumulation of sense transcripts in *Mu killer* individuals that failed to silence *MuDR* elements. Third, the impact of *Mu killer* silencing activity on the anther transcriptome is different from the impact of spontaneous silencing.

Standard *MuDR* elements contain promoters and transcription start sites within the TIRs (at about +165 and at sequences just internal to TIRA) that generate sense *mudrA* and *mudrB* RNA while antisense transcripts result from termination failure in the region separating these two genes (Walbot and Rudenko, 2002). Transgenic maize expressing additional antisense RNA failed to suppress *MuDR* sense transcript accumulation (Kim and Walbot, 2003); nonetheless, in this study a single copy *Mu killer* element producing 0.74 and 0.65 kb antisense *mudrA* transcripts was associated with effective silencing. Because these transcripts terminate in the flanking *amy4* locus, it is possible that antisense RNA stability or effectiveness depends on the sequences contributed by the flanking gene. Both antisense transcripts are complementary to a short region of about 250 bases in authentic *mudrA* transcripts, including part of the second exon and the first exon including the 5′ untranslated region. Because the 0.65 and 0.74 kb RNAs cannot form large hairpin structures, it is presumed that small RNAs derived from them target either translation or transcription of *mudrA*. This is a new model to explain how *Mu killer* silences *MuDR*. It is possible that the minor longer transcript types (Figure 6) also contribute through the previously proposed mechanism of long hairpin formation (Slotkin et al., 2005).

In *Mu killer* individuals that failed to silence *MuDR*, we found sense rather than antisense transcripts derived from *Mu killer*. These read through transcripts initiated in the *amy4* flanking locus, and also included the non-transcribed portion of TIRA, and *mudrA*-homologous sequences. A complex splicing pattern was observed, utilizing canonical 5′ donor and 3′ acceptor sites that are not used in authentic *mudrA* transcripts (Figure 7). Because *MuDR* is transcribed independent of its insertion sites, it is thought that transcription at *MuDR* loci is driven primarily by the enhancer elements located within the element TIRs; there is no evidence for read through transcripts when *MuDR* is inserted within the coding region of *bz2-mu4* (Hershberger et al., 1995) although the TIRs function as promoters when assayed in heterologous constructs (Benito and Walbot, 1994; Raizada et al., 2001a,b). Thus the unusual structure of *Mu killer* and the flanking sequence context unexpectedly highlight that both *MuDR* promoters and the insulator functions of the TIRs can be suppressed.

If there is an “arms race” between transposon and host mechanisms to suppress them, aberrant transposon forms could be a potent weapon in host-mediated defenses as originally proposed for *Mu killer*. Endogenous *hMuDR* elements reside in every maize line tested; they are co-linear with *MuDR* but contain point mutations and other small defects that make them incompetent to program *Mu* element activities. They are also impotent in regulating *MuDR* (Rudenko and Walbot, 2001) suggesting that in this case *MuDR* persisted, or won the contest. The newly arisen *Mu killer* element can silence *MuDR* with reasonable frequency; however, we also found that when *Mu killer* fails to silence *MuDR* it switches from producing antisense to producing sense *mudrA* transcripts. In this case too, it appears that *MuDR* is victorious. The stability of the *Mu killer* transcription states is unknown, but it will be interesting in future studies to assess the persistence of *Mu killer* over a multiple generation time scale to determine if, like *hMuDR* elements, it ultimately fails to contain *MuDR/Mu* elements.

## Conflict of Interest Statement

The authors declare that the research was conducted in the absence of any commercial or financial relationships that could be construed as a potential conflict of interest.

## Footnotes

^1^http://www.ncbi.nlm.nih.gov/geo/

^2^http://maizecyc.maizegdb.org/overviewsWeb/celOv.shtml

^3^http://bioinformatics.ai.sri.com/ptools/
